# Book Review: Successful Aging: A Neuroscientist Explores the Power and Potential of Our Lives

**DOI:** 10.3389/fpsyg.2021.705368

**Published:** 2021-06-18

**Authors:** Alex Siu Wing Chan, Jacqueline Mei Chi Ho, Hon Lon Tam, Patrick Ming Kuen Tang

**Affiliations:** ^1^Department of Applied Social Sciences, The Hong Kong Polytechnic University, Hong Kong, China; ^2^School of Nursing, The Hong Kong Polytechnic University, Hong Kong, China; ^3^Education Department, Kiang Wu Nursing College of Macau, Macao, China; ^4^State Key Laboratory of Translational Oncology, Department of Anatomical and Cellular Pathology, The Chinese University of Hong Kong, Hong Kong, China

**Keywords:** aging & well-being people, healthy aging, neuroscience (psychology), successful aging, psychological health

Successful aging has developed into a critical term for defining the standard of aging. The definition evolved from a biomedical perspective to include a broader view of late-life cultural and mental adjustment mechanisms. Nevertheless, there is no universally accepted description of healthy aging. Authentic scientific publications have a small following compared to books promising the mysteries of optimal health; Levitin, a legitimate researcher, seeks to combine the finest of all worlds. Although several of his recommendations are familiar, he places a premium on proper research, which means audiences will discover life-extension strategies backed by research (albeit not in human populations). Although the writer cautions against common misinformation, he provides a wide array of supplements, behavioral changes, and medical devices backed up by nothing more than rational hypotheses or colleagues obsessed with health. Levitin has provided a clear description of how the nerve and muscle work. His meticulously written piece debunks persistent misconceptions about the certainty of intellectual loss by providing insightful analysis of how our early year's memories, attitudes, social interactions, and habits all influence our brain's growth. Speaking in favor of older adults, Levitin demonstrates how everyone could improve their ability to remain alert, joyful, and intelligent while they get older. Even though researchers have yet to establish a standardized definition of “healthy aging,” one thing is certain: everyone has the potential to lead a fruitful senior life. This revelation is especially pertinent to contemporary society. This is because, traditionally, people desired longevity, but modern society has advanced to the point where people value health in their later years just as much. As such, this book offers value to every contemporary reader.

In the book's introduction, Levitin defined aging solely as a failing: a failing in the body, the mind, and even the spirit. The idea of healthy aging has generated much controversy in the latest days (Borras et al., 2020), and different aspects of the notion have been proposed in multiple analyses. Levitin explained that successful aging establishes a new way of thinking about our final decades. Using various disciplines, this book demonstrates in the introductory part that aging is not simply a period of decay but a unique developmental stage that, like infancy or adolescence, has its own set of demands and advantages.

Like Levitin, Rowe, and Kahn identify successful aging as biological solid, mental, and relational performance in late adulthood and free from severe illnesses. The primary objective of the philosophy of successful aging is to maximize the number of balanced and active years of one's existence (Plugge, 2021). In part 2, Levitin noted that the environmental factors discussed here could positively or negatively impact how we perceive old age, our engagement with the world, our habits, our will to live, and medicine. The developmental aspect of successful aging is a second strand of the narrative, which begins, ironically, in childhood. Levitin explained that we could make modifications so that aging is as enjoyable as it can be—perhaps becoming the best part of your life. The improvements are not all that difficult to implement (p. 233).

Successful aging could be seen from a community's or an individual standpoint (Esteves et al., 2020). In terms of the community, it is characterized by health factors and engagement to promote strategies. At the same time, for an individual, it is defined by health, biological, and mental function and social engagement. Since healthy aging is a highly complex term encompassing realms of physical, functional, cultural, and mental well-being, it is necessary to include all aspects while analyzing the process, both empirical and personal circumstances had (Lee et al., 2020). In other words, it would not be possible to yield meaningful results solely by analyzing the individual or the community as there are multiple factors associated with the overall health of the older adults (Chan, 2021a,b).

This book demonstrates that attitude is a significant element of a pleasant, fruitful existence and may genuinely alter our character. In Chapter 4, Levitin mentioned the healthy practices of the COACH principle are partly responsible for people with increased health spans: Curiosity, Openness, Associations, Conscientiousness, and Healthy practices. Conscientiousness and willingness to practice are numerically the two most significant trait characteristics associated with healthy aging. Conscientiousness is a set of parts that pertain to trustworthiness, honesty, fulfilling your promises, and being conscientious. Conscientiousness is a character attribute identified as a “tendency to respond in certain ways under certain circumstances” or, more broadly, the proclivity to perceive, sound, and act in a reasonably stable and constant manner over time in trait-enabling contexts (Pocnet et al., 2020). Therefore, according to this section, conscientiousness enables us to complete tasks, such as obtaining a Pilates trainer and showing up (p. 118). When ill, a diligent individual contacts a physician and accepts it when the physician specifies medicine. Many people may not be grateful for these things. A responsible citizen avoids living above their income and saves unexpected events or retirement resources. Therefore, the book explained that any of these factors contribute to a balanced and prolonged existence.

Flexibility entails a willingness to experiment and an enthusiasm for fresh concepts and methods of performing tasks. That becomes more relevant when we mature since we have a natural inclination to avoid doing different ideas and instead stick to what we have already known, resulting in a more significant intellectual loss. In part 2, Levitin mentioned we simply ought to remain conscious and combat arrogance to achieve the same result. It is critical to connecting ourselves with strangers, especially the younger generation, and experiment with fresh concepts. It does not have to be a risky activity, but a novel one. When it comes to conscientiousness, often a specific occurrence can force you to change, such as getting diagnosed with diabetes and being forced to adjust your diet or perish (chapter 9). Numerous cognitive and physical benefits have been observed with various diets, including the dash, mind, and Mediterranean diets. The Mediterranean diet (Med-Diet) was first defined by Ancel Keys as low in saturated fat and high in vegetable oils, as observed in Greece and Southern Italy during the 1960s (Martínez-González and Sánchez-Villegas, 2004). Still, there is little evidence to support these claims. The putative causes of cognitive decline and Alzheimer's are oxidative stress, inflammation of neural tissue, and vascular problems caused by the buildup of harmful substances in the circulatory system (p. 266–268). On the other hand, the book is an excellent discussion since all of us are aware that exercising is beneficial to our inner world. To say it another way, physical and mental fitness go hand in hand. However, this book in chapter 10 suggests specific strategies to ensure that the human brain stays fresh, such as mountaineering, which involves navigation abilities. Levitin stated that robotic training is beneficial because he enjoys increasing his heartbeat and oxygenating the body. That means exercise is helpful for the nervous system; however, this is primarily about cardiac well-being (p. 294).

Numerous older people experience discomfort, some of which are permanent. Levitin explains in this book that the way we experience pain is partially dictated by what the feeling represents to us. Levitin states that persistent suffering that seems to have little cause and that you cannot alleviate is devastating. However, Levitin clarified how to practice meditation in Chapter 3—Buddhist monks and those who practice mindfulness have resolved this (p. 364). This does not imply that it is no longer painful; nevertheless, we will get to a stage where it does not irritate you. Indeed, the latest evidence demonstrated that monks who had practice meditation for over 20,000 h could mentally brace for imminent suffering and remain unaffected by all this. Everyone should do a kind of mind training, yoga, mindfulness, or whatever fits us, as suggested by Levitin. This book listed some types of unbearable pain, and many patients suffer from persistent pain, which may be debilitating. The reality is that therapy is notoriously ineffective at curing debilitating pain. It is a critical barrier that we must overcome. As the author notes in the book, a large amount of support for medical science is directed toward extending human life, not toward boosting human well-being or happiness. Therefore, when discussing cognitive performance in this book, the criterion is an individual's capacity to act generally in everyday lives. This involves making sound judgments, resolving conflicts, communicating effectively with others, and maintaining a mental equilibrium (Hachinski et al., 2021). In chapter 3, Levitin highlighted the hippocampus, the cognitive system that facilitates memory, evolved for geo navigation, assisting us in remembering where we are moving. The author suggested that we travel toward good things while avoiding risk; we ignore the aspect at our peril if we do not keep it active. The hippocampus is susceptible to decay. Getting outdoors is beneficial, and it is possible as long as we commit to it (p. 94). Levitin thus suggested that to maintain a certain level of vigilance, much of this is essential for the brain to remain vibrant.

“Successful Aging: A Neuroscientist explores the power and potential of our lives” outlined a highly effective method to achieve successful aging. Older individuals have accumulated more knowledge and experiences just by their longevity (Chan et al., 2021). This results in an expanded capacity for trend extraction, for recognizing connections between environments can result in an improved judgment and problem-solving. I strongly recommend this book because it serves as a reminder to people that being conscientious, being receptive to growth possibilities, maintaining engaging relationships with everyone, particularly with the younger generation, being enthusiastic, and adhering to healthier activities, which involve food, decent sleep, and exercise are always critical.

## Author Contributions

This whole book review is prepared by AC, JH, and HT have given a valuable comment. PT has given clinical pieces of advice. All authors contributed to the article and approved the submitted version.

## Conflict of Interest

The authors declare that the research was conducted in the absence of any commercial or financial relationships that could be construed as a potential conflict of interest.
